# Cesium based phasing of macromolecules: a general easy to use approach for solving the phase problem

**DOI:** 10.1038/s41598-021-95186-1

**Published:** 2021-08-23

**Authors:** Wolfgang Koelmel, Jochen Kuper, Caroline Kisker

**Affiliations:** Rudolf Virchow Center, University of Würzburg, Würzburg, Germany

**Keywords:** Structural biology, X-ray crystallography

## Abstract

Over the last decades the phase problem in macromolecular x-ray crystallography has become more controllable as methods and approaches have diversified and improved. However, solving the phase problem is still one of the biggest obstacles on the way of successfully determining a crystal structure. To overcome this caveat, we have utilized the anomalous scattering properties of the heavy alkali metal cesium. We investigated the introduction of cesium in form of cesium chloride during the three major steps of protein treatment in crystallography: purification, crystallization, and cryo-protection. We derived a step-wise procedure encompassing a “quick-soak”-only approach and a combined approach of CsCl supplement during purification and cryo-protection. This procedure was successfully applied on two different proteins: (i) Lysozyme and (ii) as a proof of principle, a construct consisting of the PH domain of the TFIIH subunit p62 from *Chaetomium thermophilum* for de novo structure determination. Usage of CsCl thus provides a versatile, general, easy to use, and low cost phasing strategy.

## Introduction

Solving the phase problem is an essential step in the determination of any structure by X-ray crystallography^1^. Several methods have been developed to approach the phase problem for biological macromolecules. If good homology models exists, phasing is often straightforward by applying molecular replacement (MR)^2–4^.With an increasing number of structural models deposited into the protein data bank, the impact of MR for phasing is steadily increasing. However, for the determination of new protein folds or distant homologues, MR cannot be applied. In these cases, a de novo phasing strategy is inevitable. In general, five techniques can be distinguished: single wavelength anomalous diffraction (SAD)^5–7^, multi wavelength anomalous diffraction (MAD)^8,9^, macromolecular ab initio phasing^10^, multiple isomorphous replacement (MIR)^11^, and single isomorphous replacement (SIR)^11,12^. The latter two can be combined with anomalous scattering (MIRAS, SIRAS)^13,14^. Nowadays the most commonly used de novo phasing method is SAD^15^. SAD generally requires the presence of ordered heavy atoms, which can act as anomalous scatterers. It exploits the differences in the intensity of the Bijvoet Pairs caused by the imaginary f'' component of the anomalous scattering. From these differences, the positions of the anomalous scatterers can be derived and this substructure can then be used to solve the phase problem. A commonly used SAD method is based on the substitution of methionine with seleno-methionine during protein expression^16,17^. This method however, requires a suitable expression system and the presence of sufficient methionines in the macromolecule. In addition, the natively present sulfur in cysteine and methionine can be exploited for SAD (S-SAD)^18,19^, but this approach is also limited to proteins containing a sufficient amount of cysteines and methionines. Furthermore, high resolution is required as the anomalous signal of sulfur is quite small^20^. These restrictions call for alternative approaches that mildly introduce anomalous scatterers which can be exploited for phasing.

**Figure 1:**
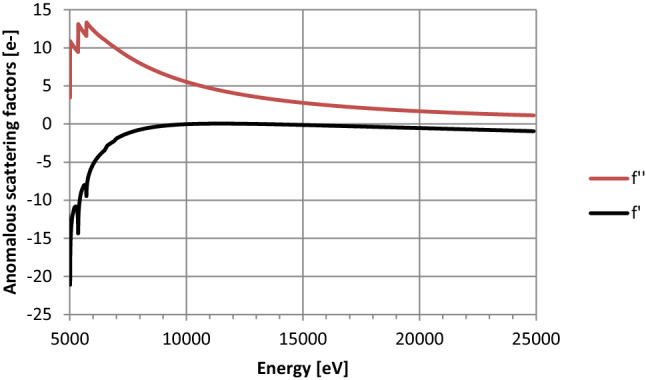
Anomalous scattering of cesium plotted against energy. The plot was generated with data from^27^.

Here, we investigated the introduction of an anomalous scatterer at different stages of the crystallization process, i.e. during protein purification, protein crystallization, and crystal cryo-protection. This harbours the potential for a step-wise procedure. The first and simplest procedure would be a “quick-soak” strategy, using already existing crystals^21^. In case this strategy doesn’t yield the means for solving the phase problem, an anomalous scatterer could be introduced at an earlier stage i.e. during protein purification or crystallization. This approach requires the identification of a suitable compound, which is compatible with each of these stages. Commonly used components within protein buffers are sodium chloride (NaCl) or potassium chloride (KCl). These salts are also used in crystallization solutions, serving as precipitant or additive to aid crystallization^22,23^. Na and K are both alkali metals, and the group is completed by rubidium (Rb), cesium (Cs), and francium (Fr). Since elements from the same group of the periodic system of the elements usually display similar chemical properties, we chose Cs as a possible candidate. Cs is the heaviest, not radioactive member of this group with very potent anomalous scattering propensities (Fig. 1). Indeed, Cs has been utilized to overcome the phase problem in prior studies^24,25^, and has been proposed as general phasing strategy in RNA crystallography^26^. To validate the approach, we pursued our analysis with two different proteins. First, hen egg white lysozyme (HEWL) to validate the general applicability. Second, a construct of the pleckstrin homology (PH) domain of the TFIIH subunit p62 from *Chaetomium thermophilum* (p62 PH), encompassing the first 109 residues. The structure of this target has so far not been determined by other means.

## Results

### HEWL crystallization and phasing

**Figure 2:**
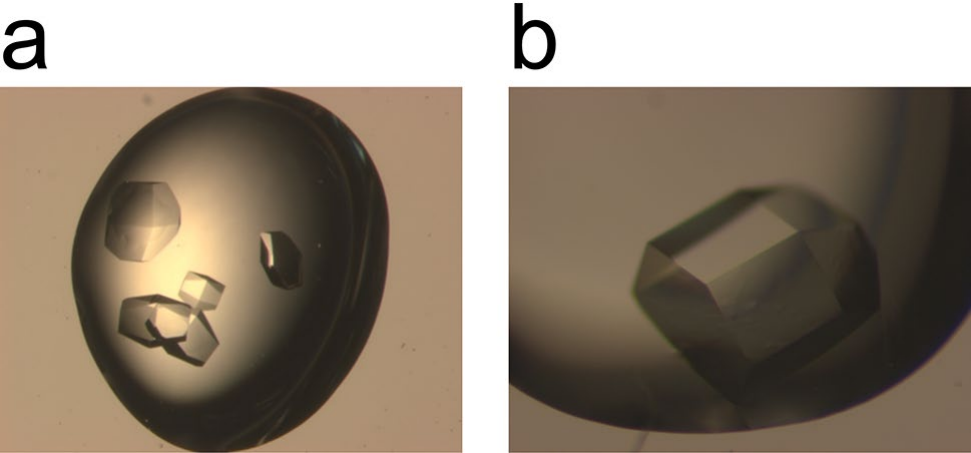
Crystallization of HEWL. (**a**) HEWL crystallized in the presence of NaCl. (**b**) HEWL crystallized in the presence of CsCl.

**Figure 3:**
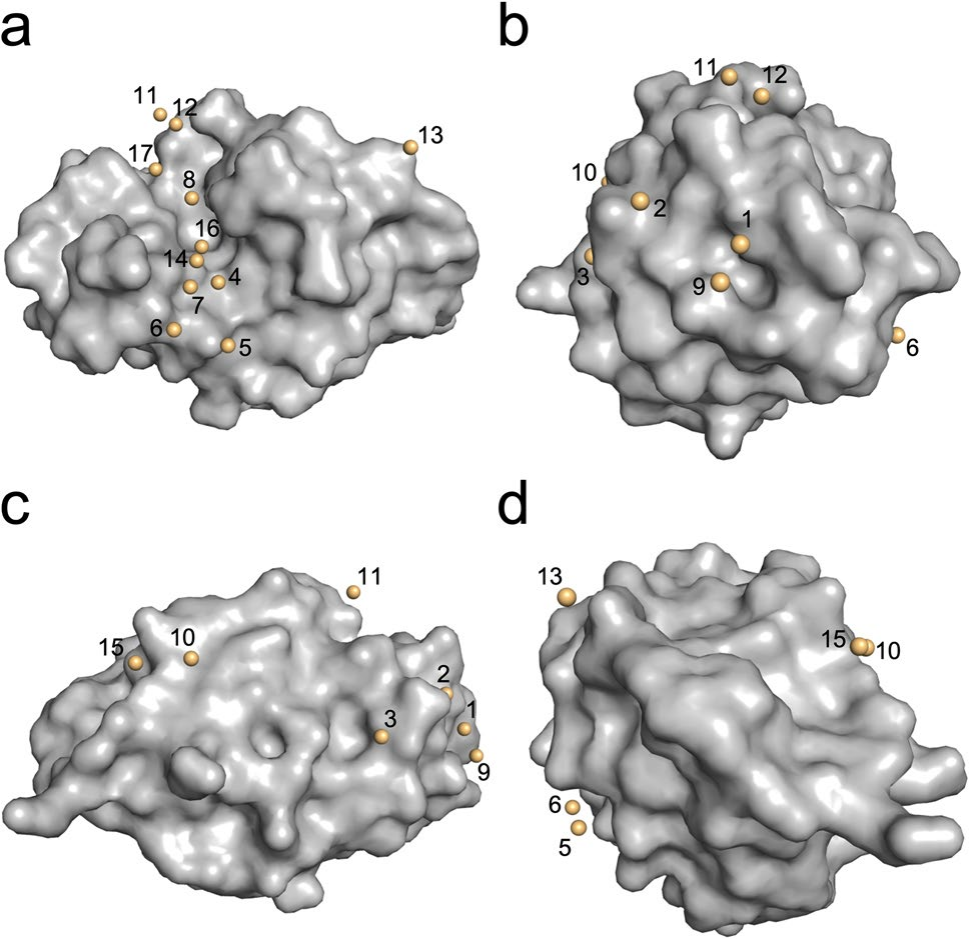
Overview of all observed cesium sites in HEWL. Numbering is coherent with Table 5. HEWL is represented as surface, cesium ions as spheres. (**a**) Front view. (**b**) Side view left. (**c**) Back view. (**d**) Side view right.

As a proof of concept for the feasibility of our strategy, we initiated our analysis with different approaches to HEWL crystallization. As outlined above, we introduced CsCl at different stages in the crystallization process. Starting with the substitution of standard buffer components like KCl or NaCl we supplemented HEWL with 0.25 M CsCl, a salt concentration that is commonly used in protein buffers for crystallization. Additionally, we supplemented CsCl in the crystallization buffer and/or the cryo-protectant solution (see Table 1). In the case of HEWL the addition of CsCl did not affect crystal growth in any of the evaluated approaches. Crystal morphology or the space group were not affected either indicating no major impact on protein quality or crystallization behaviour (Fig. 2). Subsequently, data sets were collected from the crystals obtained from these different approaches to compare the feasibility and success rate. All approaches led to crystals that could be phased using the anomalous signal as described in the methods section. All cesium sites that were identified during the different approaches have been numbered and are depicted in Fig. 3.

**Table 1:** Crystallization and cryo-protectant conditions of HEWL crystals.

#	Protein buffer	Crystallization	Cryo-protectant
1	0.25 M CsCl	50 mM NaAc pH 4.5 1.71 M NaCl	50 mM NaAc pH 4.5 1.71 M NaCl 15% (v/v) EG^a^
2	0.25 M CsCl	50 mM NaAc pH 4.5 1.71 M NaCl	50 mM NaAc pH 4.5 0.25 M CsCl 1.46 M NaCl 15% (v/v) EG
3	H_2_O	50 mM NaAc pH 4.5 1.71 M NaCl	50 mM NaAc pH 4.5 1.71 M CsCl 15% (v/v) EG
4	0.25 M CsCl	50 mM NaAc pH 4.5 1.71 M NaCl	50 mM NaAc pH 4.5 1.71 M CsCl 15% (v/v) EG
5	H_2_O	50 mM NaAc pH 4.5 1.5 M CsCl	50 mM NaAc pH 4.5 1.5 M CsCl 20% (v/v) EG

^a^Ethylene glycol.

**Figure 4:**
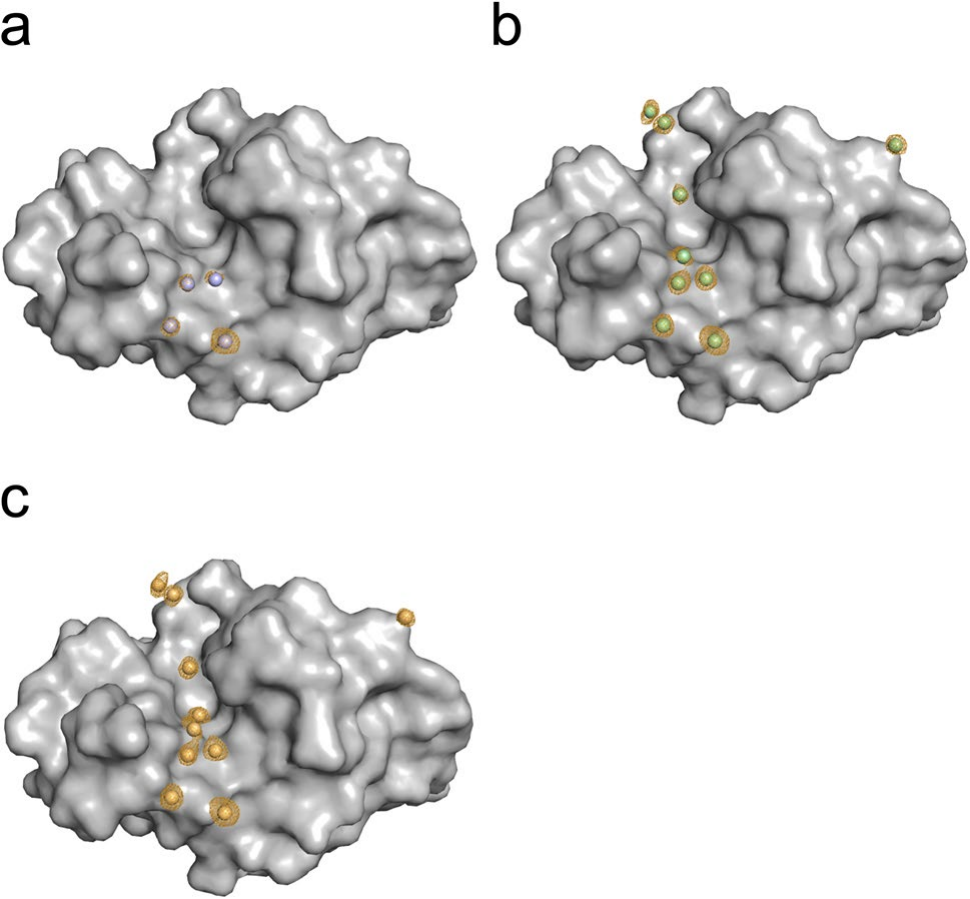
Cesium substructure of HEWL after supplementing with CsCl. HEWL is represented as grey surface, cesium ions are represented as spheres. Orange meshes display the anomalous density contoured at 3 σ. (**a**) Dataset #2: Protein dissolved in a solution containing 0.25 M CsCl and supplemented with 0.25 M CsCl in the cryo-protectant solution. (**b**) Dataset #3: Protein dissolved in H_2_O and supplemented with 1.71 M CsCl in the cryo-protectant solution. (**c**) Dataset #4: Protein dissolved in a solution containing 0.25 M CsCl and supplemented with 1.71 M CsCl in the cryo-protectant solution.

The cesium substructure, after supplementing with CsCl at the different stages towards crystallization, is depicted in Fig. 4. The substructure of CsCl only present in the protein buffer is not shown, as no bound cesium ions could be observed. The data collection and refinement statistics and the statistics for the different steps in the structure solution process provided by the Crank2 pipeline are shown in Tables 2, 3 and 4, respectively. The occurrence and occupancies of the cesium sites for all HEWL datasets are summarized in Table 5.

**Table 2:** Data collection and refinement statistics of HEWL datasets.

#	1(7BMO)	2(7BMP)	3(7BMQ)	4(7BMR)	5(7BMS)
Beamline	P14 (PETRAIII)	ID29 (ESRF)	P14 (PETRAIII)	P14 (PETRAIII)	ID29 (ESRF)
Wavelength (Å)	1.7712	1.7712	1.7712	1.7712	1.71075
Space group	P 4_3_2_1_2	P 4_3_2_1_2	P 4_3_2_1_2	P 4_3_2_1_2	P 4_3_2_1_2
**Unit cell parameters**					
a/b/c (Å)	78.63/78.63/36.76	78.95/78.95/36.87	78.75/78.75/36.81	78.97/78.97/36.81	75.93/75.93/35.71
α/β/γ (°)	90/90/90	90/90/90	90/90/90	90/90/90	90/90/90
Resolution range (Å)	39.31–1.90 (1.94–1.90)	39.48–1.90 (1.94–1.90)	39.38–1.79 (1.82–1.79)	39.48–1.78 (1.82–1.78)	37.96–1.75 (1.78–1.75)
R_merge_	0.061 (1.650)	0.057 (0.174)	0.104 (0.452)	0.152 (0.460)	0.157 (0.421)
R_pim_	0.013 (0.454)	0.012 (0.039)	0.023 (0.147)	0.033 (0.155)	0.033 (0.166)
Observed reflections	187,204 (8017)	214,048 (12,322)	221,409 (4371)	222,086 (4158)	223,578 (4113)
Unique reflections	9509 (582)	9678 (619)	11,383 (502)	11,475 (508)	10,988 (556)
<I/σI>	35.8 (1.5)	43.4 (17.0)	24.9 (4.5)	18.1 (4.5)	18.3 (4.5)
CC_1/2_	1.0 (0.684)	1.0 (0.994)	0.999 (0.881)	0.996 (0.882)	0.996 (0.902)
Completeness (%)	99.5 (96.1)	100.0 (100.0)	98.7 (78.6)	98.9 (80.4)	99.6 (95.2)
Multiplicity	19.7 (13.8)	22.1 (19.9)	19.5 (8.7)	19.4 (8.2)	20.3 (7.4)
Completeness anomalous (%)	99.2 (96.2)	99.9 (100)	98.1 (68.7)	98.3 (71.2)	99.5 (92.9)
RMS correlation ratio anomalous (low/high)	1.58 (2.44/1.01)	1.73 (4.25/0.94)	2.7 (5.98/1.06)	1.97 (4.55/1.19)	1.55 (2.9/1–1)
Multiplicity anomalous	10.6 (7.3)	12.2 (10.6)	10.4 (5.0)	10.3 (4.7)	10.9 (3.8)
Resolution range (Å)	39.31–1.90	39.48–1.90	39.38–1.79	39.48–1.78	37.96–1.75
Number of reflections	9017	9175	10,537	10,889	10,370
Number of atoms	1176	1178	1171	1178	1187
R_work_	0.208	0.155	0.209	0.177	0.175
R_free_	0.254	0.195	0.257	0.212	0.209
Mean B-factor (Å^2^)	23.441	18.865	18.518	17.969	16.969
**RMS deviations**					
Bond lengths (Å)	0.014	0.018	0.018	0.02	0.028
Bond angles (°)	1.572	1.79	1.784	2.04	2.005
**Ramachandran statistics (%)**					
Favored	99.21	97.64	98.43	99.21	98.43
Allowed	0.79	2.36	1.57	0.79	1.57
Outliers	0	0	0	0	0

Statistics for the highest resolution shell are given in parentheses.

**Table 3:** Phasing procedure for HEWL datasets.

#	Phasing method	Substructure determination	Initial phases	Density modification	Automated model building
1	SAD	SHELXC/SHELXD	refmac5	parrot	Buccaneer
2	SAD	SHELXC/SHELXD	refmac5	parrot	Buccaneer
3	SAD	SHELXC/SHELXD	refmac5	parrot	Buccaneer
4	SAD	SHELXC/SHELXD	refmac5	parrot	Buccaneer
5	SAD	SHELXC/SHELXD	refmac5	parrot	Buccaneer

**Table 4:** Phasing and structure solution data for HEWL.

	Sites/above 0.25	Substructure determination	FOM initial phases all data	Density modification	Automated model building FOM/residues built %	Final refinement R/Rfree
1	14	CFOM 53.5	0.20	0.30	0.79/91	0.36/0.40
2	14	CFOM 54.1	0.31	0.43	0.77/100	0.33/0.37
3	12	CFOM 66.5	0.25	0.46	0.76/100	0.36/0.42
4	11	CFOM 50.0	0.31	0.41	0.89/100	0.27/0.32
5	11	CFOM 49.1	0.26	0.54	0.88/94	0.34/0.35

Data collection strategy was optimized for S-SAD.

Table 5 shows that cesium sites could only be observed when CsCl was used in the buffer and was at least present in the cryo-protectant. The anomalous signal of the data where CsCl is present in the experiment also improves supporting that Cs is incorporated at stable positions (Table2). In line with this the HEWL dataset #1 in which no Cs site could be detected shows the smallest anomalous signal as indicated by the RCR (Rms Correlation Ratio) anomalous. However, this dataset could still be solved using the standardized approach indicating that the sulfur signal was picked up for successful phasing. The phasing success rate and the statistics given at each step for the Crank2 pipeline (Table 4) support the observation that Cs incorporation is beneficial for phasing HEWL. We observe a clear step and concentration dependent effect of CsCl in the figure of merit (FOM) derived from the initial phases and after initial density modification (Tables 1 and 4) thus further supporting that HEWL phasing has benefited from the described procedure and phasing statistics have improved as compared to dataset #1. In addition, the number of sites show a clear concentration dependent effect (Table 5). When comparing datasets #3 and #4, one additional site and a higher overall occupancy sum could be observed for the latter. In comparison to dataset #2 both, #3 and #4, are superior with respect to sites and occupancy. Incorporating CsCl at high concentrations in the crystallization condition and the cryo-protectant yields a high number of sites with the highest occupancy as indicated in dataset #5. In summary, our data indicate that CsCl is a feasible phasing option and is easily incorporated into protein structures using different approaches. More importantly, we could observe that CsCl is readily interchangeable with commonly used salts like KCl and NaCl. However, one caveat in this experimental approach was that the structure of HEWL could also be solved using the standardised SAD procedure for Cs phasing in the absence of Cs sites indicating that the anomalous signal derived from the sulfur sites is also present in the phasing procedure for Cs thus impairing a final judgement on the feasibility of this strategy.

**Table 5:** Occurrence, occupancy and B factor of cesium sites in HEWL for the different datasets.

#	CsCl supplement			Cesium site^a^																	Sites^e^	Σ Occ^f^	Occ/site^g^
	Diss^b^	Crys^c^	Cryo^d^	1	2	3	4	5	6	7	8	9	10	11	12	13	14	15	16	17			
1	0.25	–	–																		0	0	
2	0.25	–	0.25	0.44/41.0	0.22/64.1	0.22/62.6	0.21/62.8	0.37/51.4	0.23/52.1	0.19/50.1											7	1.88	0.27
3	–	–	1.71	0.37/31.1	0.81/39.7	0.45/44.9	0.43/34.5	0.89/35.4	0.35/34.8	0.38/33.5	0.27/44.1	0.28/39.5	0.21/46.2	0.71/74.0	0.44/51.7	0.32/37.7	0.4/064.3	0.34/54.3			15	6.65	0.44
4	0.25	–	1.71	0.48/29,1	0.85/37.8	0.5/42.4	0.39/33.1	0.91/33.8	0.46/31.9	0.37/31.2	0.3236.0	0.3/36.7	0.27/40.7	0.63/69.6	0.38/50.6	0.30/38.2	0.34/51.0	0.30/38.3	0.38/46.1		16	7.18	0.45
5	–	1.5	1.5	0.32/38.6	0.84/35.2	0.29/48.4	0.4/36.5	0.77/33.3	0.21/40.1	0.35/33.6	0.28/43.3	0.29/39.8	0.36/57.8	0.54/46.1	0.45/43.9	0.33/41.3	0.32/45.5	0.38/55.7		0.56/49.0	16	6.69	0.42

^a^Occupancy/B factor of observed sites is given. Numbers correspond to sites in Fig. 3.

^b^CsCl concentration in mol/l HEWL was dissolved in.

^c^Supplement of CsCl to crystallization condition in mol/l.

^d^Supplement of CsCl to cryo-protectant in mol/l.

^e^Total number of observed sites.

^f^Sum of occupancies of all observed sites.

^g^Average occupancy per site.

### Purification and crystallization of p62 PH in the presence of CsCl

**Figure 5:**
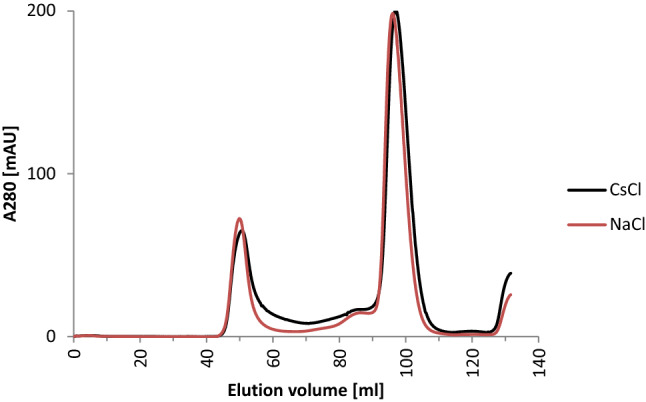
SEC elution profiles of the p62 PH domain in NaCl-buffer (red) and CsCl-buffer (black).

**Figure 6:**
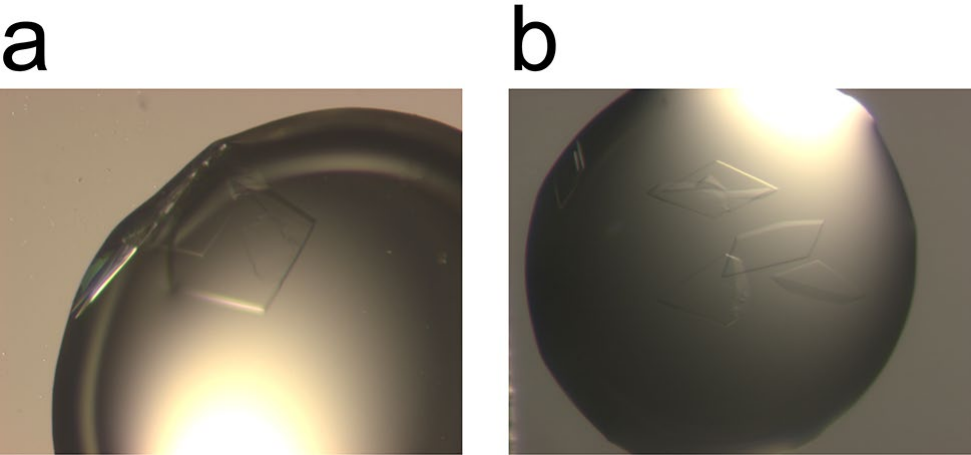
Crystallization of p62 PH. (**a**) p62 PH crystallized in KCl. (**b**) p62 PH crystallized in CsCl.

To overcome the aforementioned problem of an unbiased de novo phasing approach we applied our strategy to a novel, not yet by X-ray crystallography characterized protein. As a target for the de novo phasing approach we chose a subdomain of the p62 protein from the TFIIH complex of the eukaryote *Chaetomium thermophilum*. We cloned the pleckstrin homology domain of p62 (p62 PH) and overexpressed it in *Escherichia coli* (see methods section for details). The His-tagged protein was first purified via affinity chromatography. The subsequent size exclusion chromatography (SEC) was performed either in NaCl-buffer or in CsCl-buffer to assess whether CsCl has an effect on protein quality or oligomerisation. The elution profiles are virtually identical, revealing no significant effect when cesium was utilized instead of sodium in this step of the purification process (Fig. 5). Furthermore, the presence of CsCl in the crystallization solution did not impact crystallization as depicted in Fig. 6. Taken together, these results further support that CsCl may be highly compatible with purification and crystallization of macromolecules. The different approaches are summarised in Table 6. The final data collection and refinement statistics are provided in Table 7.

**Table 6:** Crystallization and cryo-protectant conditions of p62 PH crystals.

#	Protein buffer	Crystallization	Cryo-protectant
1	20 mM CHES pH 9.0 0.25 M NaCl	0.9 M KCl 19% (w/v) PEG 4000	0.9 M KCl 19% (w/v) PEG 4000 20% (v/v) glycerol
2	20 mM CHES pH 9.0 0.25 M CsCl	0.8 M KCl 17% (w/v) PEG 4000	0.75 M KCl 17.5% (w/v) PEG 4000 20% (v/v) glycerol
3	20 mM CHES pH 9.0 0.25 M CsCl	0.6 M KCl 15% (w/v) PEG 4000	0.25 M CsCl 0.5 M KCl 17.5% (w/v) PEG 4000 20% (v/v) glycerol
4	20 mM CHES pH 9.0 0.25 M NaCl	0.9 M KCl 17% (w/v) PEG 4000	0.75 M CsCl 17.5% (w/v) PEG 4000 20% (v/v) glycerol
5	20 mM CHES pH 9.0 0.25 M CsCl	0.9 M KCl 17% (w/v) PEG 4000	0.75 M CsCl 17.5% (w/v) PEG 4000 20% (v/v) glycerol
6	20 mM CHES pH 9.0 0.25 M NaCl	0.7 M CsCl 18% (w/v) PEG 4000	0.7 M CsCl 18% (w/v) PEG 4000 20% (v/v) glycerol

**Table 7:** Data collection and refinement statistics of p62 PH datasets.

#	1(7BMZ)	2(7BMU)	3(7BMV)	4(7BMW)	5(7BMX)	6(7BMY)
**Data collection and processing**						
Beamline	BM14 (ESRF)	P14 (PETRAIII)	ID29 (ESRF)	ID29 (ESRF)	ID29 (ESRF)	ID29 (ESRF)
Wavelength (Å)	1.7712	1.7712	1.7712	1.7712	1.7712	1.77114
Space group	C 222_1_	C 222_1_	C 222_1_	C 222_1_	C 222_1_	C 222_1_
**Unit cell parameters**						
a/b/c (Å)	34.74/106.17/76.21	34.46/106.52/75.90	39.74/102.30/75.09	40.25/101.76/75.10	34.27/106.78/75.32	34.43/106.79/75.87
α/β/γ (°)	90/90/90	90/90/90	90/90/90	90/90/90	90/90/90	90/90/90
Resolution range (Å)	43.56–2.50	75.90–1.90	42.27–1.90	42.12–1.90	43.56–1.90	43.67–1.80
	(2.60–2.50)	(1.94–1.90)	(1.94–1.90)	(1.94–1.90)	(1.94–1.90)	(1.84–1.80)
R_merge_	0.142 (1.176)	0.080 (0.450)	0.093 (1.420)	0.123 (1.392)	0.071 (0.266)	0.150 (1.359)
R_pim_	0.041 (0.473)	0.017 (0.114)	0.019 (0.296)	0.025 (0.283)	0.021 (0.080)	0.026 (0.293)
Observed reflections	63,946 (3604)	236,591 (9776)	287,034 (15,642)	300,407 (17,430)	125,536 (7410)	417,502 (10,544)
Unique reflections	5119 (521)	11,097 (643)	12,135 (692)	12,177 (739)	10,885 (658)	12,670 (487)
<I/σI>	14.4 (1.6)	28.7 (6.9)	21.6 (2.1)	17.4 (2.2)	25.5 (9.2)	22.0 (2.7)
CC_1/2_	0.997 (0.633)	0.999 (0.965)	0.999 (0.841)	0.998 (0.833)	0.999 (0.983)	0.999 (0.851)
Completeness (%)	99.1 (93.0)	97.4 (90.0)	97.8 (92.3)	97.4 (93.6)	96.3 (92.5)	94.9 (65.1)
Multiplicity	12.5 (6.9)	21.3 (15.2)	23.7 (22.6)	24.7 (23.6)	11.5 (11.3)	33.0 (21.7)
Completeness anomalous (%)	99.2 (93.2)	96.6 (89)	97.7 (92.1)	98.1 (95.6)	95.3 (90.4)	94.5 (65.5)
RMS correlation ratio anomalous (low/high)	0.99 (1.01/1.07	1.16 (1.45/1.05)	1.66 (4.05/0.97)	1.77 (3.09/0.915)	1.48 (3.36/0.90)	1.81 (3.39/1.14)
Multiplicity anomalous	6.8 (3.6)	11.0 (7.7)	12.5 (11.7)	13.1 (12.7)	5.9 (5.8)	17.0 (10.8)
**Refinement**						
Resolution range (Å)	43.56–2.50	53.26–1.90	42.27–1.90	42.12–1.90	43.56–1.90	43.67–1.80
Number of reflections	4844	10,482	11,528	11,574	10,293	12,052
Number of atoms	970	1073	944	987	1042	1101
R_work_	0.202	0.162	0.193	0.198	0.184	0.183
R_free_	0.266	0.224	0.223	0.221	0.216	0.215
Mean B-factor (Å^2^)	41.74	28.638	43.584	45.198	23.412	19.978
**RMS deviations**						
Bond lengths (Å)	0.012	0.019	0.018	0.019	0.019	0.02
Bond angles (°)	1.598	2.004	1.826	1.93	1.94	2.108
**Ramachandran statistics (%)**						
Favored	98.23	100	98.06	98.15	99.12	100
Allowed	1.77	0	1.94	1.85	0.88	0
Outliers	0	0	0	0	0	0

^a^Statistics for the highest resolution shell are given in parentheses.

### Phasing and structure solution of p62 PH

**Figure 7:**
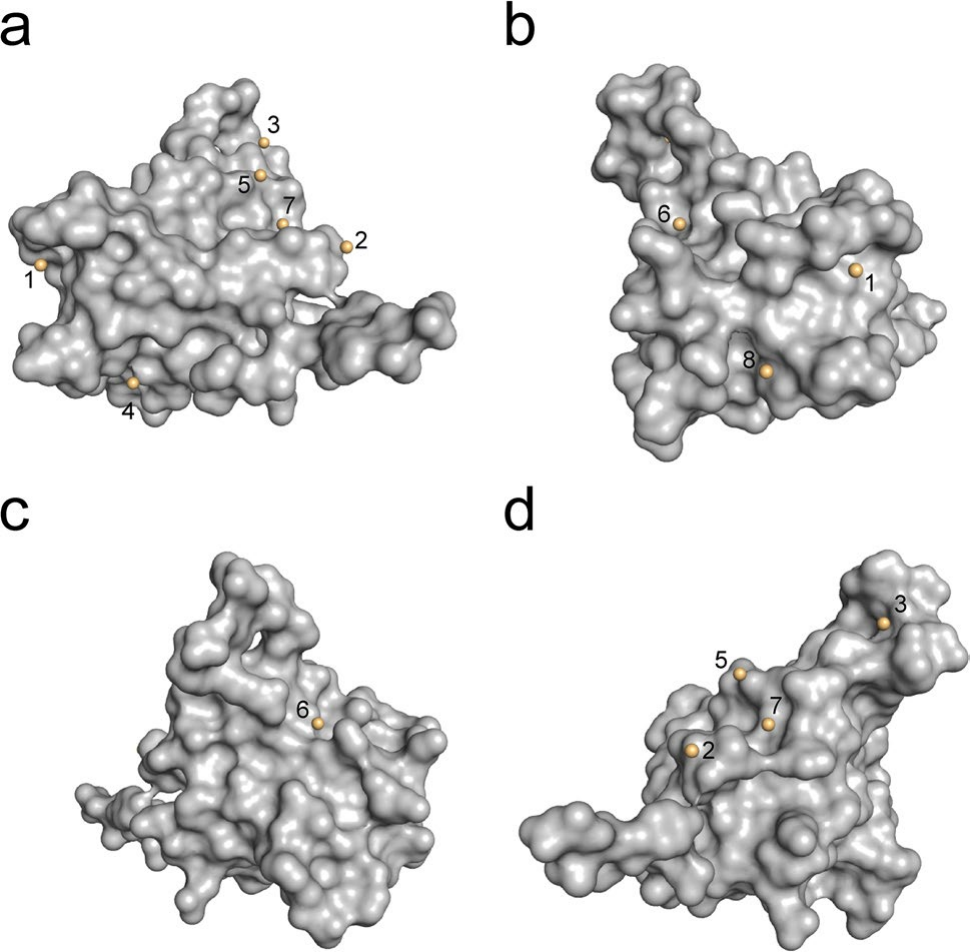
Overview of all observed cesium sites in the p62 PH domain. Numbering is coherent with Table 10. P62 PH is represented as grey surface, cesium ions as yellow spheres. (**a**) Front view. (**b**) Side view left. (**c**) Back view. (**d**) Side view right.

Using our above described strategy, we were able to solve and build the complete p62 PH protein model. We succeeded with 4 of the 6 employed approaches for phasing (Tables 6, 7, 8 and 9). The same phasing strategy as for HEWL was employed to obtain comparable results. The phasing statistics for the p62 pH domain improved with the stepwise addition of CsCl, indicating incorporation of Cs that can be harnessed during the phasing procedure. This is again reflected by the anomalous signal of the datasets representing the different approaches. The first two datasets that could not be phased experimentally show no or only a very small anomalous signal (RCR anomalous, Table 8). With the stepwise increase in CsCl concentration in the experiment, the anomalous signal increased to values between 1.5 and 1.9 as defined by the overall anomalous RMS correlation ratio given by aimless. However, the FOM derived from the initial phases did not permit a clear distinction on the success rate since only the last two datasets containing the highest concentrations of CsCl in the experimental approach showed better FOM values compared to the other datasets. After initial density modification the FOMs improved and ultimately led to successful automated structure solution. Data sets #3 and #4 only led to a significant solution after the automated model building routine was employed, suggesting that the signal that can be derived from Cs was very weak but could be utilized. To obtain an overview for all the approaches, unsuccessful de novo phasing cases were phased via rigid body refinement against models from solved datasets. All cesium sites that were observed during the different approaches (Tables 6, Table 10) have been numbered and are depicted in Fig. 7.

**Figure 8:**
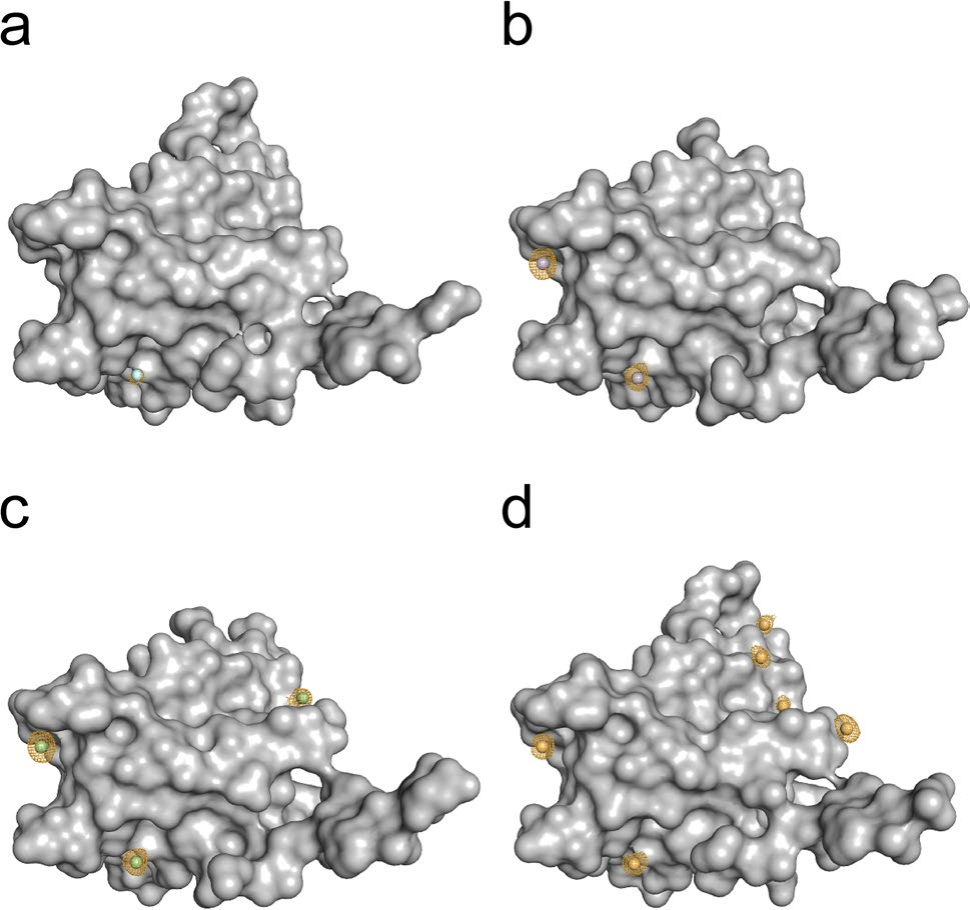
Cesium substructure of the p62 PH domain after supplementing with CsCl at different stages of the purification and crystallization process. P62 PH is represented as grey surface, cesium ions are represented as spheres. Orange meshes display the anomalous density contoured at 3 σ. (**a**) Dataset #2: Protein purified in CsCl-buffer. (**b**) Dataset #3: Protein purified in CsCl-buffer and supplemented with 0.25 M CsCl in the cryo-protectant solution. (**c**) Dataset #4: Protein purified in NaCl-buffer and supplemented with 0.75 M CsCl in the cryo-protectant solution. (**d**) Dataset #5: Protein purified in CsCl-buffer and supplemented with 0.75 M CsCl in the cryo-protectant solution.

**Figure 9:**
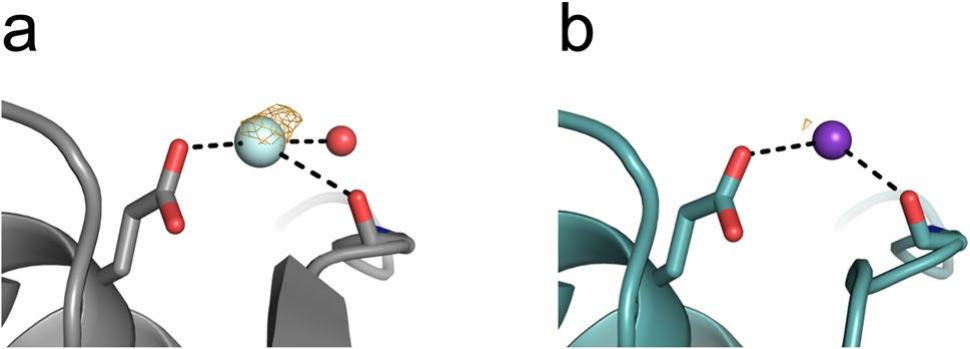
Comparison of cesium site 4 of p62 PH supplemented with CsCl only during SEC to the corresponding potassium site in p62 PH without CsCl treatment. Orange meshes display the anomalous density contoured at 3 σ. (**a**) Dataset #2: P62 PH supplemented with CsCl. (**b**) Dataset #1: P62 PH without CsCl supplement.

The cesium substructure after supplementing with CsCl at different stages of the purification and crystallization process is depicted in Fig. 8. For treatment with CsCl only during SEC (crystal #2), a low anomalous peak was observed at site 4. Compared to p62 PH without CsCl treatment (crystal #1) the anomalous peak at this site is higher for dataset #2 (Fig. 9). Thus this site was modelled as potassium in dataset #1 and a Cs in dataset#2 (Fig. 9) and the following datasets. However, the resolution of dataset #1 was lower compared to #2 (Table 7).

**Figure 10:**
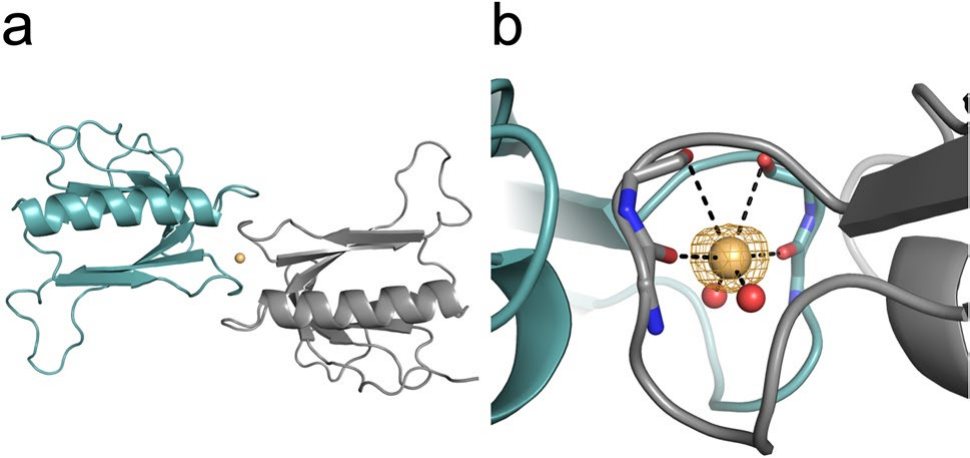
Cesium site 1 in p62 PH occupies a special position. (**a**) Cartoon representation of two p62 PH molecules related by a crystallographic two-fold axis perpendicular to the paper plane. The cesium ion located on this axis is represented as a sphere. (**b**) Detailed view of cesium site 1. The orange mesh displays the anomalous density contoured at 3 σ.

**Figure 11:**
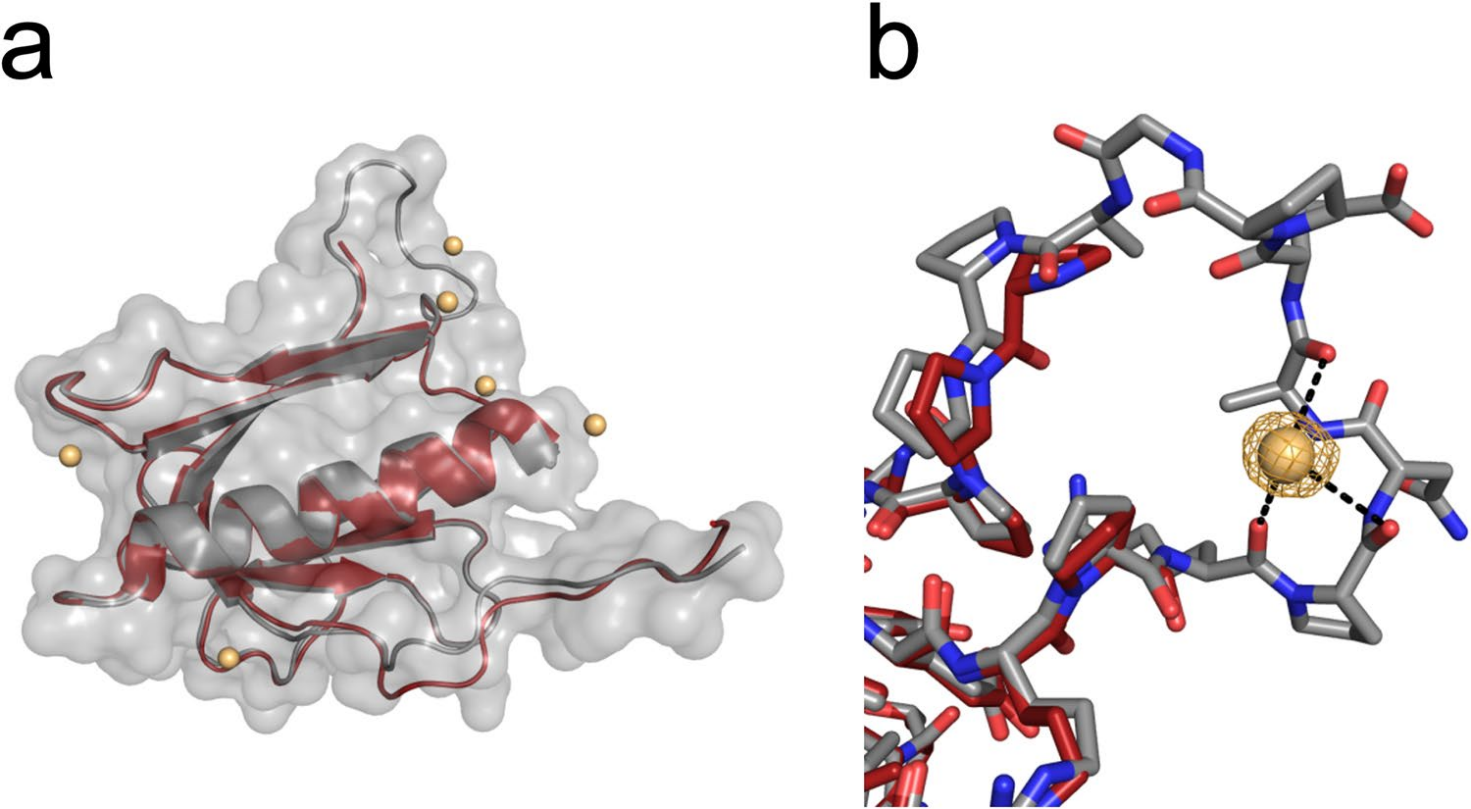
Superposition of p62 PH models from crystals with different unit cell sizes. (**a**) Surface and cartoon representation in grey correspond to dataset #5. Cesium sites are displayed as spheres. The cartoon in red corresponds to dataset #3. The loop region at the top is not present in the red model, due to disorder. (**b**) Detailed view of the loop region with cesium site 3. The orange mesh displays the anomalous density contoured at 3 σ.

The occurrence and occupancies of the final individual cesium sites for all datasets are listed in Table 10, alongside with the overall occupancy sum and average occupancy per site. Cesium site 1 poses a particular case as it lies on a special position, i.e. a crystallographic two-fold axis (Fig. 10). In this case the doubled occupancy is given. Crystals #3 and #4 display slightly different unit cell parameters (Table 7), going along with a disordered loop region for these datasets (Fig. 11a). As cesium site 3 is coordinated by this loop (Fig. 11b), this site is absent in crystals #3 and #4.

The analysis of supplementing with CsCl during the purification, crystallization, or cryo-protection process (Table 10), indicates an additive effect with respect to bound ions and overall occupancy, which is in line with the observations during the automated phasing procedure employed by the Crank2 pipeline. The comparison of datasets #4 and #5 reveals three additional cesium sites and a higher overall occupancy sum for the latter. A beneficial application of this result can be observed in dataset #3, where the CsCl supplement during SEC was combined with a lowered CsCl concentration in the cryo-protection step. As expected, this approach resulted in fewer occupied sites and a lower overall occupancy, yet this procedure was still sufficiently powerful to overcome the phase problem by means of SAD (Table 8 and 9). Importantly, in contrast to HEWL, phasing for p62 PH was only possible with the CsCl approach, whereas S-SAD alone or MR was not successful. For MR, an NMR model of the human PH domain was available as search model (PDB code: 1PFJ). Comparison of this search model with our p62 PH structure yielded an RMSD of about 2.4 Å, indicating significant deviations of both models. Especially, region 110–120 from p62 PH differs from the search model. These findings strongly emphasize the benefit of our approach for a de novo phasing strategy that is highly compatible with the purification and crystallization workflow.

## Discussion

CsCl was introduced during all three major steps of sample treatment in crystallography: purification, crystallization, and cryo-protection. No detrimental effects during SEC (Fig. 5), crystallization (Fig. 2, Fig. 6), or cryo-protection could be observed. Ultimately, de novo structure solution by means of SAD was successful employing our strategy for p62 PH (Table 6 and 9), whereas the S-SAD approaches failed. Remarkably, even low incorporation as shown for datasets #3 and #4 support structure solution. The expected electrons from Cs as compared to S at the employed wavelength (1.7712 Å) should lead to a signal for Cs that is approx. 12 times higher than for S^27^ thus permitting successful phasing with one Cs site that is only partially occupied in the case of p62 PH. The expected higher signal of Cs is reduced in all cases that we analysed since the sites were only partially occupied. However, beneficial effects for phasing can still be observed due to the much higher expected signal. The high compatibility with all three steps in protein handling renders CsCl a highly versatile compound for experimental phasing and enables a flexible adjustment of heavy atom introduction, depending on the specific needs for a particular project.

Due to its compatibility with purification, crystallization, and cryo-protection, CsCl can be used in various ways. First, usage in cryo-soaks (“quick-soaks”) as described for halides by Dauter et al.^21^ is possible, as demonstrated for p62 PH dataset #4. Cryo-soaking with cesium provides a good alternative to soaking with halides, especially when crystals suffer from halide treatment or no bound halides can be obtained due to unfavourable surface charge of the target protein. Here, the opposite charge of cesium can be beneficial. Second, supplementing with CsCl at an early step of protein handling. i.e. during SEC can be combined with cryo-soaks. For both proteins tested in this study, additive effects with respect to bound ions and overall occupancy could be observed. The boosted anomalous signal might be beneficial for difficult borderline cases. Third, this additive effect can be exploited to reduce the CsCl concentration in the cryo-protection step. This approach might be beneficial for proteins, which can only tolerate limited amounts of CsCl, as this procedure would provide much milder soaking conditions. Fourth, if NaCl or KCl are present in the crystallization condition, co-crystallization with CsCl can be conducted. Substitution of NaCl or KCl with CsCl has been successfully pursued for HEWL and p62 PH, respectively.

We therefore suggest to introduce CsCl in the work flow at the earliest possible stage i.e. at the SEC step if applicable. It remains to be investigated whether our protocol can be applied successfully to cases where significantly larger proteins need to be phased with CsCl. However, given the strong anomalous signal provided by Cs at energies that can be readily accessed at most synchrotron beamlines and the high compatibility with current protein purification and crystallization strategies application to larger proteins seems highly feasible.

The usage of CsCl provides an elegant, easy to use, and low cost phasing strategy. No special equipment is needed and the procedure can be seamlessly integrated into the common procedure of sample treatment. CsCl is broadly commercially available and much cheaper as for example seleno-methionine and the potent anomalous scattering propensities make cesium a very powerful agent for phasing. The phasing procedure with CsCl permits a flexible adjustment to the specific needs of a particular project and can be performed in a step-wise procedure.

## Methods

### Reagents, buffers, and protein preparation

The composition of the purification buffers of the p62 PH domain are the following (IMAC: immobilized metal affinity chromatography, SEC: size exclusion chromatography).

Lysis buffer: 50 mM CHES pH 9.0, 0.3 M NaCl, 5 mM Imidazole.

Elution buffer (IMAC): 50 mM CHES pH 9.0, 0.3 M NaCl, 0.25 M Imidazole.

NaCl-buffer (SEC): 20 mM CHES pH 9.0, 0.25 M NaCl.

CsCl-buffer (SEC): 20 mM CHES pH 9.0, 0.25 M CsCl.

The DNA sequence encoding the p62 PH domain from *Chaetomium thermophilum* was cloned into a pBADM-11 vector (EMBL) with an N-terminal 6 × His-tag and a TEV cleavage site. P62 PH was expressed in Arctic Express (DE3) RIL cells (Agilent). After cell harvest, the pellet was resuspended and lysed in Lysis buffer, and purified in two steps. First, IMAC was performed using Ni-TED beads (Macherey–Nagel) and bound protein was eluted with Elution buffer. Second, SEC was performed using a HiLoad 16/600 Superdex 200 pg column (Cytiva) with either NaCl-buffer or CsCl-buffer. Peak fractions were pooled and concentrated with centrifugal filter units (Merck Millipore) to 11–13 mg/ml.

HEWL was purchased as dry powder (Carl Roth) and dissolved to reach a concentration of 50 mg/ml in deionized water with 0.1 M sodium acetate pH 4.5, or 0.25 M CsCl. No further purification steps were applied.

### Crystallization

Crystallization experiments were performed using the vapor diffusion method. All solutions used for crystallization were filtered through 0.2 µm filters (Sartorius Stedim Biotech) prior to use.

Crystallization of HEWL was pursued via the hanging drop method in 24 well plates (Crystalgen). 3 µl of protein solution at a concentration of 50 mg/ml was mixed with 3 µl precipitant solution and equilibrated against 1 ml of the precipitant solution. Crystals appeared within 1 or 2 days with edge lengths mostly between 200 und 500 µm. Crystallization and cryo-protectant conditions are listed in Table 1.

Crystallization trays of p62 PH were set up via the hanging drop method in 24 well plates. 1 µl of protein solution at a concentration of 11–13 mg/ml was mixed with 1 µl precipitant solution and equilibrated against 1 ml of the precipitant solution. Plate like crystals appeared within 1 or 2 days with edge lengths mostly between 200 and 600 µm, and a thickness of 20–30 µm. Crystallization and cryo-protectant conditions are listed in Table 6.

Crystals were harvested with cryo-loops (Hampton Research) and flash frozen in liquid nitrogen.

### Data collection and processing

Data were collected via the rotation method and datasets were indexed, integrated, and scaled with XDS^28^. One dataset per crystal was collected comprising a full rotation of 360°, except for crystals #3 and #4 of p62 PH. For these, two datasets (2 full rotations of 360°) from one crystal were collected, combined, and brought to a common scale with XSCALE. Data were merged with Aimless^29^. The HEWL data for the cesium approach were collected to resolutions similar to that of the p62 PH data to obtain more comparable data for the analysis. Data collection and processing statistics are given in Tables 2 and 7 for HEWL and p62 PH, respectively.

### Structure solution and refinement

Structure solution was performed using a unified unbiased approach applying the Crank2 pipeline^30^ that is part of the current CCP4 software package. We deliberately used the default workflow without any modifications, except for the number of SHELXD trials which were raised from 2,000 to 10,000. We used the SAD pipeline that comprises the following setup: 1) Substructure detection with SHELXC,SHELXD^31^, 2) substructure phasing using refmac5^32^, 3) hand determination using solomon and multicomb, 4) density modification with parrot and refmac5, 5) automated model building with buccaneer^33^, refmac5, and parrot, and 6) model refinement using refmac5. The resolution cutoff for substructure detection that is suggested by default from SHELXC was used in all cases and ranged between 3.2 and 2.4 Å for all datasets. Phasing was performed using all data. We used 10 initial sites as estimate for the substructure search for all datasets. The structure solution procedure for each dataset is given in Tables 3 and 8 for HEWL and p62 PH, respectively. The main indicators for the quality of each step in the phasing procedure are listed in Tables 4 and 9 for HEWL and p62 PH, respectively. The structures were completed and corrected with Coot^34^. Structures were refined directly against the SAD data with refmac5. The substructure occupancy was refined as well. Model stereochemistry was analysed via the MolProbity server^35^. Refinement and model statistics are given in Tables 2 and 7 for HEWL and p62 PH, respectively. Final statistics for the Cs atoms for HEWL and p62 PH are given  in  Tables 5 and 10, respectively. (Tables 8, 9, 10).

**Table 8:** Phasing procedure for p62 PH datasets.

#	Phasing method	Substructure determination	Initial phases	Density modification	Automated model building
1	RB (#5)	–	–	–	–
2	RB^a^ (#6)	–	–	–	–
3	SAD	SHELXC/SHELXD	refmac5	Parrot	Buccaneer
4	SAD	SHELXC/SHELXD	refmac5	Parrot	Buccaneer
5	SAD	SHELXC/SHELXD	refmac5	Parrot	Buccaneer
6	SAD	SHELXC/SHELXD	refmac5	Parrot	Buccaneer

^a^Rigid body refinement using Refmac5. Numbers in parentheses indicate the starting model used for phasing.

**Table 9:** Phasing and structure solution data for p62 PH.

	Sites/above 0.25	Substructure determination	FOM initial phases all data	Density modification	Automated model building FOM/residues built %	Final refinement R/Rfree
1	7	CFOM 39.1	0.21	0.32	0.34/43	0.53/0.55
2	7	CFOM 32.1	0.16	0.39	0.45/0	0.55/0.54
3	3	CFOM 49.0	0.19	0.33	0.89/96.4	0.26/0.28
4	4	CFOM 40.1	0.20	0.33	0.89/91.7	0.27/0.32
5	10	CFOM 53.4	0.26	0.48	0.91/96.7	0.26/0.29
6	9	CFOM 49.9	0.23	0.47	0.91/98.3	0.28/0.31

**Table 10:** Occurrence, occupancy and B factors of cesium sites in p62 PH for the different datasets.

#	CsCl supplement			Cesium site^a^								Sites^f^	Σ Occ^g^	Occ/site^h^
	SEC^b^	Crys^c^	Cryo^d^	1^e^	2	3	4	5	6	7	8			
1	–	–	–									0	0	
2	0.25	–	–				0.25/69.3					1	0.25	0.25
3	0.25	–	0.25	0.72/54.5			0.36/61.3					2	1.08	0.54
4	–	–	0.75	0.9/72.2			0.41/84.7		0.27/101.7	0.42/128.7	0.2786.0	5	2.27	0.45
5	0.25	–	0.75	0.68/28.9	0.67/39.6	0.36/44.3	0.43/44.8	0.38/45.0	0.31/48.9	0.27/41.3	0.33/44.6	8	3.43	0.43
6	–	0.7	0.7	0.54/25.4	0.47/41.6	0.27/39.3	0.37/47.3	0.36/43.3	0.24/39.2	0.25/38.5	0.26/42.1	8	2.76	0.35

^a^Occupancy/B factors of observed sites is given. Numbers correspond to sites in Fig. 7.

^b^Supplement of CsCl to SEC buffer in mol/l.

^c^Supplement of CsCl to crystallization condition in mol/l.

^d^Supplement of CsCl to cryo-protectant in mol/l.

^e^Site 1 lies on a crystallographic two-fold axis (Fig. 10). Doubled occupancy is given.

^f^Total number of observed sites.

^g^Sum of occupancies of all observed sites.

^h^Average occupancy per site.

### Anomalous difference maps and final ion assignment

Anomalous difference maps were generated by directly refining against the SAD data and were used as guidance for final ion placement. Anomalous peak heights of sulfur from cysteines/methionines were used as reference to distinguish cesium from other ions. Hereby, peaks clearly exceeding the sulfur peak heights were attributed to cesium. Chloride ions were placed based on the comparison with datasets without cesium. Potassium and chloride ions were distinguished by consideration of bonding distances^36,37^.

## Data Availability

Atomic coordinates and structure factors have been deposited in the Protein Data Bank under the accession codes: 7BMO, 7BMP, 7BMQ, 7BMR, 7BMS, for hen egg white lysozyme and 7BMU, 7BMV, 7BMW, 7BMX, 7BMY, 7BMZ for ctp62.
